# Zinc Finger 521 Modulates the Nrf2-Notch Signaling Pathway in Human Ovarian Carcinoma

**DOI:** 10.3390/ijms241914755

**Published:** 2023-09-29

**Authors:** Stefania Scicchitano, Maria Concetta Faniello, Maria Mesuraca

**Affiliations:** 1 Research Center of Biochemistry and Advanced Molecular Biology, Department of Experimental and Clinical Medicine, “Magna Graecia” University of Catanzaro, 88100 Catanzaro, Italy; faniello@unicz.it; 2 Laboratory of Molecular Haematopoiesis and Stem Cell Biology, Department of Experimental and Clinical Medicine, University Magna Græcia, 88100 Catanzaro, Italy

**Keywords:** human ovarian carcinoma, NOTCH, NRF2, zinc finger protein, ZNF521

## Abstract

The human zinc finger protein 521 (ZNF521) is a co-transcriptional factor with multiple recognized regulatory functions in a range of normal, cancer and stem cell compartments. ZNF521 regulates proliferation, progression and CSC (cancer stem cell) compartments in human ovarian cancer (hOC), which is a very aggressive and late-diagnosed female tumor. Two other important regulators of hOC are the NRF2 and NOTCH signaling pathways. In the present paper, the mRNA and protein levels of ZNF521 were correlated with those of the NRF2-NOTCH signaling components in two different hOC cell lines and in a public dataset of 381 hOC patients. The data show that high levels of ZNF521 significantly increase NRF2-NOTCH signaling expression; conversely, the silencing of ZNF521 impairs NRF2-NOTCH signaling. This experimental work shows that, in hOC, different levels of ZNF521 modulate the NRF2-NOTCH signaling pathway and also influences hOC CSC properties.

## 1. Introduction

Ovarian carcinoma (OC) is the most malignant gynecological tumor [1]. A total of 75% of OC patients are diagnosed with stage III–IV cancer in the first instance, and even if a patient exhibits a good response to surgery and chemotherapy, they often exhibit tumor relapse combined with metastasis and chemoresistance [2,3]. Therefore, the identification of the molecular mechanisms and early biomarkers involved in cancer initiation, development and progression is of utmost importance in the fight against this type of tumor. Recently, we identified the multi-zinc finger protein ZNF521 as a regulator of tumor growth, proliferation and migration in hEOC (human epithelial ovarian carcinoma) through the modulation that ZNF521 exerts on key regulatory genes involved in EMT (epithelial mesenchymal transition) [4]. ZNF521 acts via multiple molecular interactions to control the homeostasis of the immature cell compartment in different tissues and in cancers [5,6,7,8,9,10,11]. ZNF521 is abundant in OC, where its high expression is associated with a poor prognosis, and a significant number of gene amplifications in the ZNF521 gene (6%) have been detected [12,13]. Indeed, ZNF521 has been included in a list of the 15 top genes associated with poor survival in patients with serous cystadenocarcinomas [14].

Several molecular pathways are involved in the development of hOC (human ovarian carcinoma), two of which are NRF2 (nuclear factor erythroid 2-related factor 2) and NOTCH signaling [15,16,17].

The transcription factor NRF2 is primarily known for its role during oxidative stress, but it is also involved in cellular metabolism, carcinogenesis and other cellular processes like inflammation and autophagy [18]. The Cap’n’Collar (CNC) subfamily of transcription factors comprises NRF1, NRF2 and NRF3 [19]. Through different conserved NRF2-ECH homology domains (Neh), NRF2 can (i) bind to ARE (antioxidant response element) sequences on its antioxidant target genes; (ii) interact with the Kelch domain of Kelch-like-ECH-associated protein 1 (KEAP1); (iii) function as a transcriptional activator; (iv) bind to E3 ubiquitin ligase and RXR (retinoic X receptor) alpha. Normally, the majority of NRF2 proteins interact with KEAP1 via Neh2 [18]. This interaction results in NRF2 ubiquitination and degradation, but during cellular stress conditions, ROS (reactive oxygen species), which play an important role in tumorigenesis [20,21,22], interfere with KEAP1, promoting NRF2 release and its translocation into the nucleus [23,24]. In oncological patients, NRF2 is associated with poor overall survival (OS). NRF2 prevents complete EMT with a more stem-like phenotype inducing chemoresistance and enhancing metastasis [25,26,27,28]. In a retrospective study on 108 OC patients, high expression of *Nrf2* was indicative of shorter disease-free survival (DFS) and overall survival (OS) [29].

The Notch pathway plays an important role in different biological processes such as the development of many organs, tissue homeostasis, cell fate and apoptosis [30,31,32,33,34]. The Notch family are single-pass transmembrane receptors that transduce signals to neighboring cells [35]. In mammals, there are four Notch receptors (Notch 1–4), activated by the ligand on adjacent cells [36,37]. Binding with a ligand leads to a conformational change and exposure of a cleavage site in its extracellular domain that moves into the nucleus and interacts with CSL (CBF1-Suppressor of Hairless-LAG1) and the Mastermind co-activator (MAML1) to activate transcription [38,39]. When this complex, containing CSL, NICD (Notch intracellular domain) and MAML1, is formed, it recruits several co-activators and co-repressors (including histone acetylase P300 and P53), which bind DNA and control the transcription of Notch target genes [40,41,42]. The Notch gene is considered an oncogene and is responsible for the formation and progression of several types of tumors [43]. The overexpression and gene amplification of Notch have been associated with hematopoietic and solid tumors including ovarian cancer [44,45,46,47,48,49,50,51,52]. The increased expression of Notch1 and of its NICD in human ovarian carcinoma induces an advantage for growth, cell proliferation and colony formation, and it is related to cancer progression, resistance to chemotherapy and a decreased survival rate, highlighting Notch as a possible therapeutic target [47,53,54,55,56].

In the present work, we modulate intracellular ZNF521 levels via lentiviral overexpression or silencing in two different hOC cell lines and demonstrate through mRNA and protein analysis that the NRF2-NOTCH axis is similarly modulated.

## 2. Results

### 2.1. ZNF521 Overexpression Modifies the NRF2-NOTCH Axis

To study the role of ZNF521 in hOC, two cell lines, HeyA8 and ES-2, were infected using a lentiviral vector expressing ZNF521, and a panel of genes involved in the NRF2-NOTCH axis was analyzed. 

qRT-PCR analysis demonstrated, in both cell lines, that the overexpression of ZNF521 increased the mRNA levels of P300, an important co-activator of NRF2 and NOTCH signaling pathways. It is known that Zfp521 (the murine homolog of human ZNF521) interacts with P300 via the ZF1 to ZF8 at its N-terminus [57].

**Figure 1:**
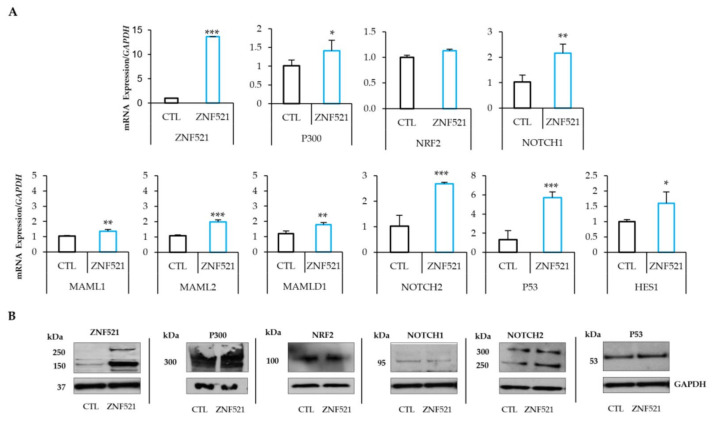
ZNF521 modulates NRF2-NOTCH1 crosstalk in HeyA8 hOC cell line. mRNA ((**A**): qRT-PCR) and protein levels ((**B**): Western blotting) of P300, NRF2 and NOTCH components in ZNF521-overexpressing HeyA8 hOC cell line in adherent cell growth conditions. CTL: empty vector. Densitometric analysis of Western blotting were shown in Figure S1A. All experiments were performed in triplicate. Asterisks indicate *p* < 0.05 *, *p* < 0.01 **, *p* < 0.001 ***.

**Figure 2:**
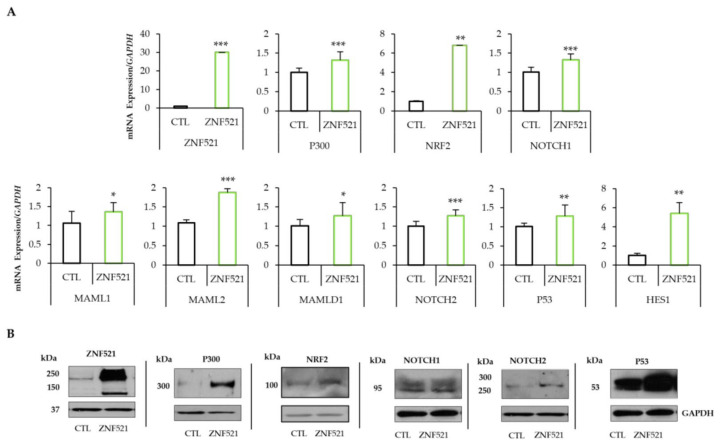
ZNF521 modulates NRF2–NOTCH1 crosstalk in ES-2 hOC cell line. mRNA ((**A**): qRT-PCR) and protein levels ((**B**): Western blotting) of P300, NRF2 and NOTCH components in a ZNF521-overexpressing ES-2 hOC cell line in adherent cell growth conditions. CTL: empty vector. Densitometric analysis of Western blotting were shown in Figure S1B. All experiments were performed in triplicate. Asterisks indicate *p* < 0.05 *, *p* < 0.01 **, *p* < 0.001 ***.

In our models, ZNF521 enhanced the transcription levels of *P300* and *NOTCH1* in both cell lines, but only in ES-2 cells were the *NRF2* transcripts also significantly modulated (Figure 1A and Figure 2A). This modulation was confirmed at the protein level, where P300, NRF2 and NOTCH components (NOTCH1, NOTCH2, MAML1, MAML2, MAMLD1, P53 and HES1) were analyzed. The results showed that the NRF2-NOTCH axis was induced by ZNF521, via P300, only in the ES-2 hOC cell line (Figure 1B: HeyA8 and Figure 2B: ES-2). In particular, the expression levels of *NOTCH1*, *NOTCH2*, *MAML2*, *P53* and *HES1* were upregulated 1.84- to 5.31-fold, as well as a small increase in MAML1 and MAMLD1, which were upregulated by 1.41 to 1.5-fold in both transduced human OC cell lines. Moreover, the expression of *P300*, even if high at basal level, was upregulated (more significantly in ES-2 than in HeyA8 cells) by ZNF521 (Figure 1A and Figure 2A). In ES-2 cells, the modulated expression of *P300* enhanced NRF2 levels 6.79-fold. The results illustrated in Figure 1 and Figure 2 show that the overexpression of ZNF521 significantly amplified the expression of NRF2 and NOTCH. These data were confirmed via western blotting (Figure 1B and Figure 2B) and prompted us to investigate the effects of ZNF521 on the NRF2-NOTCH pathway only in ES-2 cells.

### 2.2. Nrf2-Notch Axis was Modulated by ZNF521 also in 3D Culture

**Figure 3:**
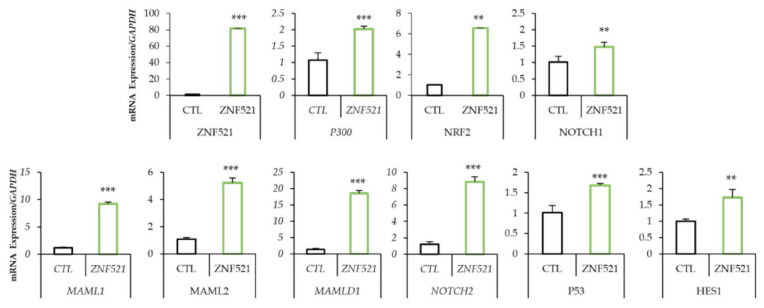
ZNF521 modulates NRF2-NOTCH signaling in anchorage-independent growth. Modulation of the NRF2-NOTCH axis by ZNF521 overexpression in an ES-2 hOC cell line: mRNA expression levels by qRT-PCR (A). CTL: empty vector. Densitometric analysis of Western blotting were shown in Figure S1C. All experiments were performed in triplicate. Asterisks indicate *p* < 0.01 **, *p* < 0.001 ***.

We previously demonstrated that the overexpression of ZNF521 enhances the proliferation of ES-2 cells both in dependent- and independent-anchorage conditions where ZNF521 induced an enrichment in the CSC subpopulation [4]. For this reason, ES-2 cells cultured as spheres were also investigated. The results in Figure 3 show that *ZNF521* induced an increase of 2-fold in the transcript levels of *P300* and of 6.54-fold in *NRF2* levels compared to control cells. This modulation largely resulted in an overall upregulation trend of the NOTCH pathway. In particular, mRNA transcripts for *NOTCH2*, *MAML1*, *MAML2* and *MAMLD1* increased 5- to 18-fold compared to 2-fold for the *HES1 NOTCH* target gene. 

### 2.3. Silencing of ZNF521 Impairs the ES-2 Spheroid Formation Ability

**Figure 4:**
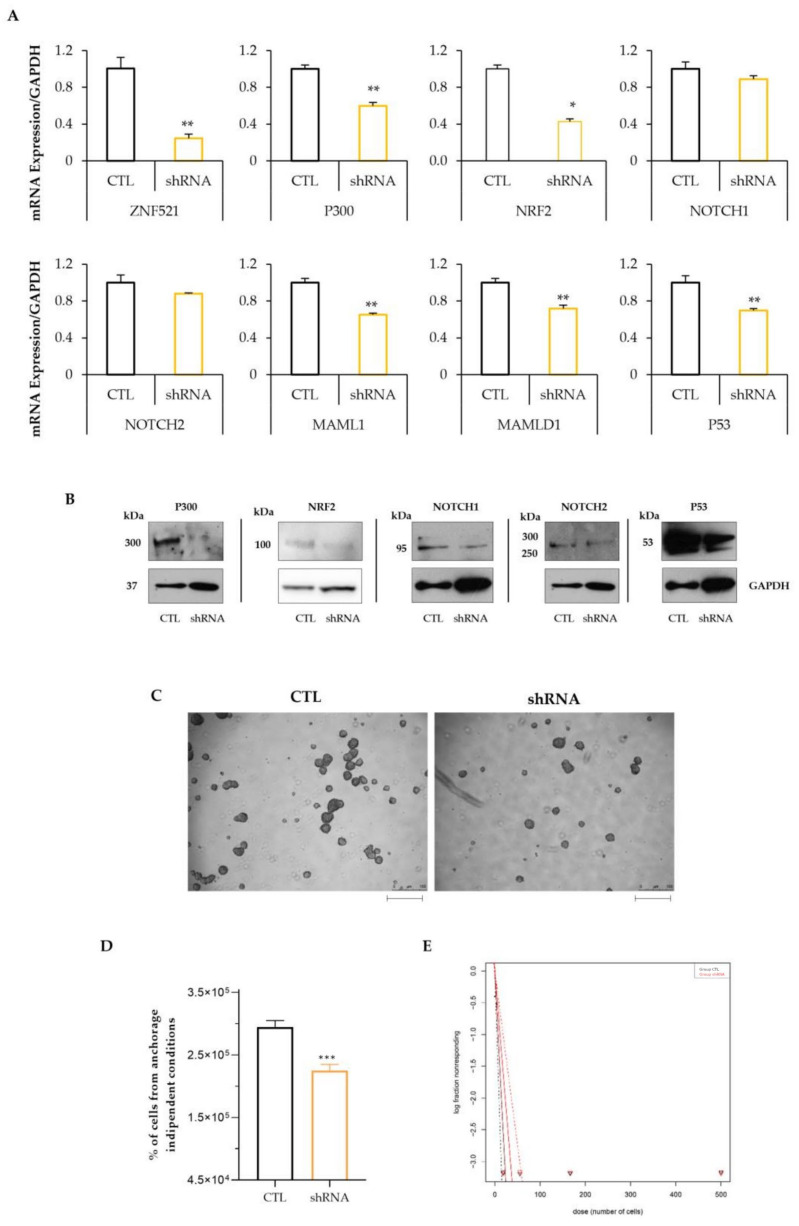
Effect of ZNF521 knockdown by shRNA in the ES-2 cell line. Silencing of ZNF521 in adherent cell growth conditions (**A**: first graph). Modulation of NRF2-NOTCH axis: qRT-PCR (**A**) and Western blotting (**B**). Silencing of ZNF521 reduced the sphere-forming ability in ES-2 cells: representative images (5×) are shown (**C**); the number of cells derived from spheres were counted, as shown in (**D**), and analyzed with ELDA software version 5.6.1.5980 of 01.06.2023 (**E**). CTL: non-target shRNA control vector; shRNA: shRNA lentiviral vector for silencing of ZNF521 expression. Scale bars correspond to 100 µm. All experiments were performed in triplicate. Asterisks indicate *p* < 0.05 *, *p* < 0.01 **, *p* < 0.001 ***.

To assess the importance of ZNF521 in the NRF2-NOTCH axis for the maintenance of CSCs, ZNF521 expression was silenced in ES-2 cells. Silencing ZNF521 using a shRNA resulted in the down-regulation of the mRNA expression levels of *P300*, *NRF2* and *NOTCH* components (Figure 4A) and of the corresponding protein levels (Figure 4B). The impairment of NRF2-NOTCH signaling negatively affected the sphere formation ability of ES-2 cells. In ZNF521-silenced cells, a lower number of spheres was observed compared to control cells (Figure 4C,D). To further investigate the CSC properties, a sphere assay was performed in limiting dilution conditions (LD) and analyzed using Extreme Limiting Dilution Analysis (ELDA) software version 5.6.1.5980 of 01.06.2023 [58,59]. The results showed impairments of the stem cell frequency (1 cell/(stem cell frequency)) of 7.29 for CTL and 11.54 for shRNA cells (*p* = 0.0123). 

### 2.4. Analysis of hOC Data Set

**Figure 5:**
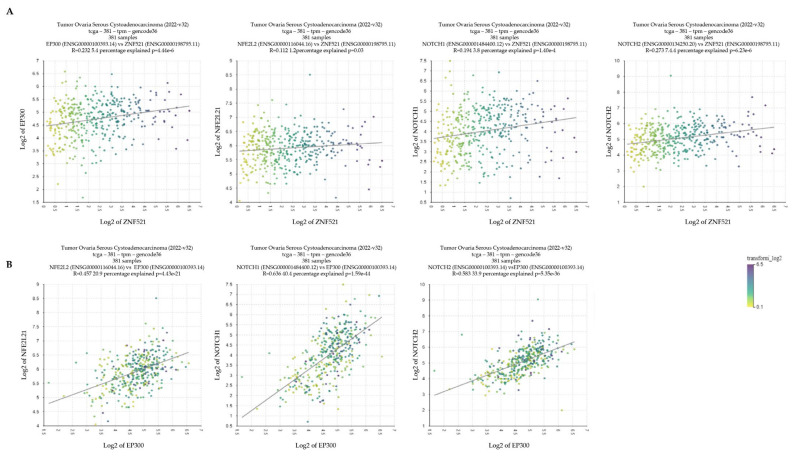
Correlation analysis between ZNF521 and the NRF2-NOTCH axis in hOC. R2 analysis of 381 specimens in a hOC public database (Tumor Ovarian Serous Cystoadenocarcinoma 2022-v32): ZNF521 expression data plotted vs. those of P300, NRF2 (NFE2L2 in figure), NOTCH1 and NOTCH2 (**A**); P300 expression data plotted vs. those of NRF2 (NFE2L2 in figure), NOTCH1 and NOTCH2 (**B**).

To establish whether there was a relationship between ZNF521 expression and NRF2-NOTCH signaling, a set of 381 hOC cases (R2 analysis platform, public database Tumor Ovarian Serous Cystoadenocarcinoma 2022-v32) [60] was analyzed. To this end, the mRNA levels of P300, NRF2, NOTCH1 and NOTCH2 were plotted against those of ZNF521 and among each other (Figure 5). The scatter profile XY plots show that the expression of P300, NOTCH1 and NOTCH2 was significantly and positively associated with the presence of a ZNF521 transcript (Figure 5A), and that of P300 was significantly and positively correlated with NRF2 (NFE2L2 in Figure 5) and NOTCH1/2 (Figure 5B).

## 3. Discussion

Understanding the molecular mechanisms involved in tumor development and progression is essential for identifying early functional and prognostic markers to implement adequate and timely cancer therapeutic strategies. Different molecular pathways are involved in the initiation and progression of human ovarian carcinoma, which is still one of the most lethal types of cancer in women [2,3,61]. ZNF521 is a well-known co-transcriptional factor involved in the homeostasis of normal, cancer and stem cell compartments. ZNF521 can enhance cellular proliferation in different tissues, reduce the cellular differentiation of neural and bone stem cells, and augment stem- and cancer stem-cell compartments [6,7,9,10,57,62,63,64,65]. In hOC, ZNF521 is highly expressed (commonly amplified), and it is one of the top genes associated with a poor prognosis and drug resistance [13,14]. In human epithelial ovarian carcinoma, ZNF521 is also considered an important regulator of the CSC compartment and EMT [4], in which two other important genes are involved: *NRF2* and *NOTCH*. In cancer, NRF2 and NOTCH signaling pathways regulate initiation, differentiation, the CSC compartment and drug resistance [25,43,66,67]. NRF2 and NOTCH signaling influence each other and are considered a unique pathway called the NRF2-NOTCH axis [68,69,70,71]. The NRF2-NOTCH axis is important for the maintenance of cellular homeostasis: it is involved in cell fate determination during hematopoiesis, and it regulates self-renewal in the lungs, adult neurogenesis occurring in the subventricular zone and osteogenesis in the bone [71,72,73,74,75,76,77,78]. In lung and hepatocellular carcinoma, the activation of the NOTCH pathway increases the expression of NRF2 and its target [69,71]. Moreover, NRF2-NOTCH signaling coordinates cancer cell migration during EMT [68]. This is not only functional crosstalk; NRF2 and NOTCH1 physically interact with each other. *NOTCH1* can bind to functional *Rbpjk* sequences present in the regulatory region of *NRF2* and, vice versa, *NRF2* can bind to a functional *ARE* sequence present in the gene regulatory region of *NOTCH1* [71]. In the NRF2 and NOTCH pathways, P300 is a functional and physical co-activator [40,41,79]. Ganner and colleagues demonstrated that P300 competes with KEAP1 for binding to NRF2 and that the overexpression of P300 significantly enhances NRF2 levels. The acetylation of NRF2 by P300 enhances the half-life of NRF2 and prevents the NRF2 degradation induced by KEAP1, increasing *NRF2* DNA binding to *ARE* target sequences (including that present on *NOTCH*) [79].

The data illustrated so far provide evidence that the overexpression of ZNF521 in HeyA8 and ES-2 human ovarian cancer cell lines induces a clear increase in the expression of NRF2 (only in the ES-2 cell line) and NOTCH component genes (Figure 1A and Figure 2A), as well as that of the corresponding proteins (Figure 1B and Figure 2B), both in 2D cultures and in non-anchorage-dependent cultures (Figure 3). On the contrary, the silencing of ZNF521 impairs P300, NRF2 and NOTCH components both at the mRNA and protein level (Figure 4A,B). This down-regulation negatively affects the ES-2 CSC subpopulation (Figure 4C,D). Additional analysis of a public database containing 381 hOC specimens [57,58] validates our in vitro results: ZNF521 expression data strongly correlate with those of the NRF2-NOTCH pathway (Figure 5).

The data shown in the present work confirm our hypothesis that ZNF521 activates the NRF2-NOTCH axis through the formation of a complex with P300 [57] that directly activates NRF2 and NOTCH [40,41,79], and also justify why they cannot be further augmented by ZNF521 overexpression in HeyA8 OC cells (where basal P300 protein levels are high).

Our data, for the first time, identified the modulatory function that ZNF521 exerts on the NRF2-NOTCH axis in this tumor model, and whose expression could be used to select hOC patients potentially responsive to treatments with NRF2 or NOTCH inhibitors [80,81]. Further analyses will help us to better characterize the molecular interactions underlying this modulation that may be the key molecular mechanism regulating the initiation, proliferation and progression of human ovarian cancer.

## 4. Materials and Methods

### 4.1. Cell Lines and Culture Conditions

The HeyA8 cell line, derived from differentiated papillary human ovary cystoadenocarcinoma, was cultured in DMEM. The ES-2 cell line, a human ovarian adenocarcinoma cell line, was cultured in RPMI. Cell culture media were supplemented with 10% fetal bovine serum, 50 U of penicillin and 50 μg of streptomycin/mL (Thermo Fisher Scientific, Milan, Italy), and cell lines were maintained at 37 °C in 5% CO_2_. 

### 4.2. Transfection and Transduction of Cell Lines

Lentiviral vectors were used to transfect HEK293T cells where 10 µg of plasmid (control vector, ZNF521-overexpressing vector or shRNA lentiviral vectors for specific silencing of ZNF521, respectively named CTL, ZNF521 and shRNA in figures) (Sigma, Milan, Italy). Each vector was added to cells with lentiviral packaging plasmids (2 µg of pCMV-VSVG and 10 µg of pCMV-deltaR8-9) and cells were transfected using the calcium phosphate method. Three rounds of transduction were performed, each for 24 h, by means of centrifuging HeyA8 or ES-2 with the lentivirus supernatant with 6 μg/mL of polybrene at 3200 rpm at 32 °C for 50 min.

The transduction was performed in three independent experiments, and cells were further sorted for EGFP, giving a homogeneous population that was over 90% positive for EGFP.

### 4.3. Sphere-Forming Assay

Transduced single cell preparations were counted and resuspended in a medium containing DMEM F12 (GIBCO, Milan, Italy), L-glutamine (Thermo Fisher Scientific), 1%, Pen/Strep (Thermo Fisher Scientific), 1%, B27 (GIBCO) 50×, 20 ng/mL hEGF (PeproTech, DBA, Milan, Italy) and 2 ng/mL hFGFb (PeproTech). ES-2 sphere medium was supplemented with 10 µg/mL of insulin (Sigma-Aldrich, Milan, Italy) and 4 μg/mL of heparin (Sigma-Aldrich) and plated at a concentration of 4.5 × 10^4^ cells/well in 6 ultra-low attachment wells (Corning Inc., Milan, Italy).

After 7 days, when spheres were observed, the number of cells for each culture was calculated, and the size of spheres was estimated from the acquired images (at 10× magnification) using ImageJ 1.51j8. All the experiments were performed in triplicate.

### 4.4. Spheres Limiting Diluitions Assay (LDA) and Extreme Limiting Dilution Analysis (ELDA)

Sphere assays were also performed under limiting dilution conditions. ES-2 cells silenced for ZNF521 were plated in 96-well ultra-low attachment plates using serial dilutions. Transduced cells were counted and plated in the appropriate medium (see paragraph 4.3) at the following concentrations: 500, 166.66, 55.55, 18.51, 6.17 and 2.05 cells/well. After 7 days, spheres were counted and analyzed using ELDA software: version 5.6.1.5980 of 1 June 2023 [58,59] to compare the enrichment or depletion in CSC populations between the CTL and shRNA transduced cells. All the experiments were performed twelve-fold.

### 4.5. Expression Analysis by qRT-PCR

A total of 1 µg of RNA, previously prepared with Tri Reagent (Sigma-Aldrich) and verified using a NanoDrop 2000/2000c Spectrophotometer (Thermo Fisher Scientific), was used to synthesize cDNA using SuperScript III reverse transcriptase and was amplified with the iQ™ SYBR^®^ green super mix (BioRad, Milan, Italy) using the qRT-PCR amplifier QuantStudio3 (Applied Biosystems, Milan, Italy).

The analysis of gene expression was calculated as 2^−ddCt^ and normalized for the house-keeping gene (GAPDH). Primers used in this study were as follows (5′–3′):

*h-ZNF521* was previously described [11];
*h-NRF2 (fwd) CACCACCCACACAACTTACTGC*,
*h-NRF2 (rev) GGTCTTCTTGGGGCTTAGGT*;
*h-NOTCH1 (fwd) CTGGAGGACCTCATCAACTC*,
*h-NOTCH1 (rev) TTCTTCAGGAGCACAACTGC*;
*h-NOTCH2 (fwd) ATGCTCAGCCGGGATACCT*,
*h-NOTCH2 (rev) GGTTGGCCACAGTGGTACAGG*;
*h-MAML1 (fwd) GCAACAGCAGTTCCTTCAGAGG*,
*h-MAML1 (rev) GTGAACTGTCCAACCTGCTGTG*.
*h-MAML2 (fwd) TGCCCAATCTCTACCAAGCCAG*,
*h-MAML2 (rev) AGCAGGGGTTAGGACTTGGACT*;
*h-MAMLD1 (fwd) CCTCAGATTCCATGCCTGCTCT*,
*h-MAMLD1 (rev) CTTGCCTT-GATCCGGCTACACTTGG*;
*h-P300 (fwd) GATGACCTTCCCAGCCTCAAA*,
*h-P300 (rev) GCCAGATGATCTCATGGTGAAGG*;
*h-P53 (fwd) CCTCAGCATCTTATCCGAGTGG*,
*h-P53 (rev) TGGATGGTGGTACAGTCAGAGC*;
*h-HES1 (fwd) CCAAAGACAGCATCTGAGCA*,
*h-HES1 (rev) GCCGCGAGCTATCTTTCTT*;
*h-GAPDH (fwd) CACCATCTTCCAGGAGCGAG*,
*h-GAPDH (rev) TCAC-GCCACAGTTTCCCGGA*.

### 4.6. Protein Extraction and Western Blotting

Nuclear proteins were obtained using a hypotonic lysis buffer consisting of 10 mM HEPES pH7.9, 10 mM KCl, 0.1 mM EDTA, protease inhibitors (P8849, Sigma-Aldrich) and phosphatase inhibitor cocktails 2 and 3 (P0044, P5726, Sigma-Aldrich), which was used to incubate cells on ice for 20 min. After the addition of 0.25% Igepal-630 (NP40) (Sigma-Aldrich), samples were centrifuged at 3000 rpm for 5 min. Nuclear pellets were resuspended in 20 mM HEPES pH7.9, 0.4 M NaCl and 1 mM EDTA with protease and phosphatase inhibitors. After three cycles of vortexing and incubation on ice, samples were centrifuged at 12,000 rpm for 20 min, and the nuclear extracts were collected.

The total protein of transduced cells was extracted as described in [82]. Proteins were denatured, reduced and separated using 4–12% NuPAGE Novex bis-Tris or 3–8% NuPAGE Tris-Acetate Protein gradient polyacrylamide gels (Thermo Fisher Scientific) and blotted onto nitrocellulose. ZNF521 was detected using a rabbit anti-ZNF521 (PA534388, Life Technologies) antibody at 1:1000, P300 with a rabbit anti-P300(C-20) (sc-585, Santa Cruz Biotechnology) antibody at 1:10000, NRF2 with a rabbit anti-NRF2 (D1Z9C) XP (#12721, Cell Signaling) antibody, NOTCH1 with a rabbit anti-NOTCH1 (ab-52301, AbCam, Milan, Italy) antibody at 1:500, NOTCH2 with a rabbit anti-NOTCH2 (sc-5545, Santa Cruz Bio-technology) antibody at 1:1000, P53 with a rabbit anti-P53 (#9282, Cell Signaling) antibody at 1:1000 and GADPH with a mouse anti-GAPDH (sc-166574 Santa Cruz Biotechnology) antibody at 1:1000. Secondary rabbit and mouse HRP antibodies were used, and signals were detected using the ImmunoCruz Western blotting luminal reagent (sc-2004, sc-2005, Santa Cruz, Biotechnology) and exposure to auto-radiographic film (GE Healthcare, Milan, Italy).

### 4.7. R2: Genomics Analysis and Visualization Platform of TCGA hOC

A public database [R2: Genomics Analysis and Visualization Platform (http://r2.amc.nl, accessed on 15 November 2015) [60] of human tumor ovarian serous cystadenocarcinoma that includes the expression data for 381 hOC specimens (Tumor Ovarian Serous Cystadenocarcinoma 2022-v32-tcga-381-tpm-gencode36) was investigated for the correlation between the mRNA of ZNF521 and the NRF2-NOTCH pathway.

### 4.8. Statistical Analysis

Students’ *t* test, assuming unequal variances between two samples, was used to determine the significant differences (*p* < 0.05 *, *p* < 0.01 **, *p* < 0.001. ***, *p* > 0.0001 ****).

## Data Availability

Data are contained within the article or Supplementary Materials.

## Supplementary Materials

The following supporting information can be downloaded at: https://www.mdpi.com/article/10.3390/ijms241914755/s1.

## References

[1] Yin M., Yang J., Zhou H., Liu Q., Li S., Zhang X. (2022). Impact of Adjuvant Chemotherapy on FIGO Stage I Ovarian Clear Cell Carcinoma: A Systematic Review and Meta-Analysis. Front. Oncol..

[2] McMullen M., Karakasis K., Rottapel R., Oza A.M. (2021). Advances in ovarian cancer, from biology to treatment. Nat. Cancer.

[3] Reid B.M., Permuth J.B., Sellers T.A. (2017). Epidemiology of ovarian cancer: A review. Cancer Biol. Med..

[4] Scicchitano S., Montalcini Y., Lucchino V., Melocchi V., Gigantino V., Chiarella E., Bianchi F., Weisz A., Mesuraca M. (2022). Enhanced ZNF521 expression induces an aggressive phenotype in human ovarian carcinoma cell lines. PLoS ONE.

[5] Mesuraca M., Chiarella E., Scicchitano S., Codispoti B., Giordano M., Nappo G., Bond H.M., Morrone G. (2015). ZNF423 andZNF521: EBF1 Antagonists of Potential Relevance in B-Lymphoid Malignancies. Biomed. Res. Int..

[6] Chiarella E., Aloisio A., Scicchitano S., Todoerti K., Cosentino E.G., Lico D., Neri A., Amodio N., Bond H.M., Mesuraca M. (2021). ZNF521 Enhances MLLAF9-Dependent Hematopoietic Stem Cell Transformation in Acute Myeloid Leukemias by Altering theGene Expression Landscape. Int. J. Mol. Sci..

[7] Kang S., Akerblad P., Kiviranta R., Gupta R.K., Kajimura S., Griffin M.J., Min J., Baron R., Rosen E.D. (2012). Regulation of early adipose commitment by Zfp521. PLoS Biol..

[8] Wu M., Hesse E., Morvan F., Zhang J.P., Correa D., Rowe G.C., Kiviranta R., Neff L., Philbrick W.M., Horne W.C. (2009). Zfp521 antagonizes Runx2, delays osteoblast differentiation in vitro, and promotes bone formation in vivo. Bone.

[9] Hesse E., Kiviranta R., Wu M., Saito H., Yamana K., Correa D., Atfi A., Baron R. (2010). Zinc finger protein 521, a new player in bone formation. Ann. N. Y. Acad. Sci..

[10] Chiarella E., Aloisio A., Scicchitano S., Bond H.M., Mesuraca M. (2021). Regulatory Role of microRNAs Targeting the Transcription Co-Factor ZNF521 in Normal Tissues and Cancers. Int. J. Mol. Sci..

[11] Scicchitano S., Giordano M., Lucchino V., Montalcini Y., Chiarella E., Aloisio A., Codispoti B., Zoppoli P., Melocchi V., Bianchi F. (2019). The stem cell-associated transcription co-factor, ZNF521, interacts with GLI1 and GLI2 and enhances the activity of the Sonic hedgehog pathway. Cell Death Dis..

[12] Zhao F., Yu Y.Q. (2018). The prognostic roles of mRNAs of the exosomes derived from bone marrow stromal cells in common malignancies: A bioinformatic study. Onco Targets Ther..

[13] cBioPortal for Cancer Genomics. https://www.cbioportal.org.

[14] An Y., Yang Q. (2020). Development and Validation of an Immune-Related Prognostic Signature for Ovarian Cancer Based on Weighted Gene Coexpression Network Analysis. Biomed Res. Int..

[15] Liao H., Zhou Q., Zhang Z., Wang Q., Sun Y., Yi X., Feng Y. (2012). NRF2 is overexpressed in ovarian epithelial carcinoma and is regulated by gonadotrophin and sex-steroid hormones. Oncol. Rep..

[16] Li D., Hong X., Zhao F., Ci X., Zhang S. (2021). Targeting Nrf2 may reverse the drug resistance in ovarian cancer. Cancer Cell Int..

[17] Wang M., Wang J., Wang L., Wu L., Xin X. (2010). Notch1 expression correlates with tumor differentiation status in ovarian carcinoma. Med. Oncol..

[18] He F., Ru X., Wen T. (2020). NRF2, a Transcription Factor for Stress Response and Beyond. Int. J. Mol. Sci..

[19] Jaramillo M.C., Zhang D.D. (2013). The emerging role of the Nrf2-Keap1 signaling pathway in cancer. Genes. Dev..

[20] Scicchitano S., Vecchio E., Battaglia A.M., Oliverio M., Nardi M., Procopio A., Costanzo F.S., Biamonte F., Faniello M.C. (2023). The Double-Edged Sword of Oleuropein in Ovarian Cancer Cells: From Antioxidant Functions to Cytotoxic Effects. Int. J. Mol. Sci..

[21] Gauron C., Rampon C., Bouzaffour M., Ipendey E., Teillon J., Volovitch M., Vris S. (2013). Sustained production of ROS triggers compensatory proliferation and is required for regeneration to proceed. Sci. Rep..

[22] Khalil H.S., Goltsov A., Langdon S.P., Harrison D.J., Bown J., Deeni Y. (2015). Quantitative analysis of NRF2 pathway reveals key elements of the regulatory circuits underlying antioxidant response and proliferation of ovarian cancer cells. J. Biotechnol..

[23] Baird L., Lleres D., Swift S., Dinkova-Kostova A.T. (2013). Regulatory flexibility in the Nrf2-mediated stress response is conferred by conformational cycling of the Keap1-Nrf2 protein complex. Proc. Natl. Acad. Sci. USA.

[24] Itoh K., Wakabayashi N., Katoh Y., Ishii T., O’Connor T., Yamamoto M. (2003). Keap1 regulates both cytoplasmic-nuclear shuttling and degradation of Nrf2 in response to electrophiles. Genes Cells.

[25] Rojo De La Vega M., Chapman E., Zhang D.D. (2018). NRF2 and the Hallmarks of Cancer. Cancer Cell.

[26] Taguchi K., Yamamoto M. (2020). The KEAP1-NRF2 System as a Molecular Target of Cancer Treatment. Cancers.

[27] Kröger C., Afeyan A., Mraz J., Eaton E.N., Reinhardt F., Khodor Y.L., Thiru P., Bierie B., Ye X., Burge C.B. (2019). Acquisition of a Hybrid E/M State Is Essential for Tumorigenicity of Basal Breast Cancer Cells. Proc. Natl. Acad. Sci. USA.

[28] Pasani S., Sahoo S., Jolly M.K. (2020). Hybrid E/M Phenotype(s) and Stemness: A Mechanistic Connection Embedded in Network Topology. J. Clin. Med..

[29] Liew P.L., Hsu C.S., Liu W.M., Lee Y.C., Lee Y.C., Chen C.L. (2015). Prognostic and predictive values of Nrf2, Keap1, p16 and E-cadherin expression in ovarian epithelial carcinoma. Int. J. Clin. Exp. Pathol..

[30] Penton A.L., Leonard L.D., Spinner N.B. (2012). Notch signaling in human development and disease. Semin. Cell Dev. Biol..

[31] Siebel C., Lendahl U. (2017). Notch Signaling in Development, Tissue Homeostasis, and Disease. Physiol. Rev..

[32] Ohata S., Aoki R., Kinoshita S., Yamaguchi M., Tsuruoka-Kinoshita S., Tanaka H., Wada H., Watabe S., Tsuboi T., Masai I. (2011). Dual roles of Notch in regulation of apically restricted mitosis and apicobasal polarity of neuroepithelial cells. Neuron.

[33] MacGrogan D., Nus M., de la Pompa J.L. (2010). Notch signaling in cardiac development and disease. Curr. Top. Dev. Biol..

[34] Bigas A., Robert-Moreno A., Espinosa L. (2010). The Notch pathway in the developing hematopoietic system. Int. J. Dev. Biol..

[35] Pratt E.B., Wentzell J.S., Maxson J.E., Courter L., Hazelett D., Christian J.L. (2011). The cell giveth and the cell taketh away: An overview of Notch pathway activation by endocytic trafficking of ligands and receptors. Acta Histochem..

[36] Langridge P.D., Struhl G. (2017). Epsin-Dependent Ligand Endocytosis Activates Notch by Force. Cell.

[37] Yamamoto S., Charng W.L., Bellen H.J. (2010). Endocytosis and intracellular trafficking of Notch and its ligands. Curr. Top. Dev. Biol..

[38] Kopan R., Ilagan M.X. (2009). The canonical Notch signaling pathway: Unfolding the activation mechanism. Cell.

[39] Gordon W.R., Arnett K.L., Blacklow S.C.J. (2008). The molecular logic of Notch signaling-a structural and biochemical perspective. Cell Sci..

[40] Saint Just Ribeiro M., Hansson M.L., Wallberg A.E. (2007). A proline repeat domain in the Notch co-activator MAML1 is important for the p300-mediated acetylation of MAML1. Biochem. J..

[41] Bray S.J. (2016). Notch signalling in context. Nat. Rev. Mol. Cell Biol..

[42] Zhao Y., Katzman R.B., Delmolino L.M., Bhat I., Zhang Y., Gurumurthy C.B., Germaniuk-Kurowska A., Reddi H.V., Solomon A., Zeng M.S. (2007). The notch regulator MAML1 interacts with p53 and functions as a coactivator. J. Biol. Chem..

[43] Venkatesh V., Nataraj R., Thangaraj G.S., Karthikeyan M., Gnanasekaran A., Kaginelli S.B., Kuppanna G., Kallappa C.G., Basalingappa K.M. (2018). Targeting Notch signalling pathway of cancer stem cells. Stem Cell Investig..

[44] Ellisen L.W., Bird J., West D.C., Soreng A.L., Reynolds T.C., Smith S.D., Sklar J. (1991). TAN-1, the human homolog of the Drosophila notch gene, is broken by chromosomal translocations in T lymphoblastic neoplasms. Cell.

[45] Purow B.W., Haque R.M., Noel M.W., Su Q., Burdick M.J., Lee J., Sundaresan T., Pastorino S., Park J.K., Mikolaenko I. (2005). Expression of Notch-1 and its ligands, Delta-like-1 and Jagged-1, is critical for glioma cell survival and proliferation. Cancer Res..

[46] Stylianou S., Clarke R.B., Brennan K. (2006). Aberrant activation of notch signaling in human breast cancer. Cancer Res..

[47] Reedijk M., Odorcic S., Chang L., Zhang H., Miller N., McCready D.R., Lockwood G., Egan S.E. (2005). High-level coexpression of JAG1 and NOTCH1 is observed in human breast cancer and is associated with poor overall survival. Cancer Res..

[48] Guest R.V., Boulter L., Dwyer B.J., Kendall T.J., Man T.Y., Minnis-Lyons S.E., Lu W.Y., Robson A.J., Gonzalez S.F., Raven A. (2016). Notch3 drives development and progression of cholangiocarcinoma. Proc. Natl. Acad. Sci. USA.

[49] Westhoff B., Colaluca I.N., D’Ario G., Donzelli M., Tosoni D., Volorio S., Pelosi G., Spaggiari L., Mazzarol G., Viale G. (2009). Alterations of the Notch pathway in lung cancer. Proc. Natl. Acad. Sci. USA.

[50] Hopfer O., Zwahlen D., Fey M.F., Aebi S. (2005). The Notch pathway in ovarian carcinomas and adenomas. Br. J. Cancer.

[51] Silva F., Félix A., Serpa J. (2016). Functional redundancy of the Notch pathway in ovarian cancer cell lines. Oncol. Lett..

[52] Vanorny D.A., Mayo K.E. (2017). The role of Notch signaling in the mammalian ovary. Reproduction.

[53] Rose S.L., Kunnimalaiyaan M., Drenzek J., Seiler N. (2010). Notch 1 signaling is active in ovarian cancer. Gynecol. Oncol..

[54] Chen C., Wang X., Huang S., Wang L., Han L., Yu S. (2017). Prognostic roles of Notch receptor mRNA expression in human ovarian cancer. Oncotarget.

[55] Alniaimi A.N., Demorest-Hayes K., Alexander V.M., Seo S., Yang D., Rose S. (2015). Increased Notch1 Expression Is Associated With Poor Overall Survival in Patients With Ovarian Cancer. Int. J. Gynecol. Cancer.

[56] Ma Y., Wang X., Qiu C., Qin J., Wang K., Sun G., Jiang D., Li J., Wang L., Shi J. (2021). Using protein microarray to identify and evaluate autoantibodies to tumor-associated antigens in ovarian cancer. Cancer Sci..

[57] Kamiya D., Banno S., Sasai N., Ohgushi M., Inomata H., Watanabe K., Kawada M., Yakura R., Kiyonari H., Nakao K. (2011). Intrinsic transition of embryonic stem-cell differentiation into neural progenitors. Nature.

[58] ELDA: Extreme Limiting Dilution Analysis. https://bioinf.wehi.edu.au/software/elda/.

[59] Hu Y., Smyth G.K. (2009). ELDA: Extreme limiting dilution analysis for comparing depleted and enriched populations in stem cell and other assays. J. Immunol. Methods.

[60] R2: Genomics Analysis and Visualization Platform. http://r2.amc.nl.

[61] Siegel R.L., Miller K.D., Wagle N.S., Jemal A. (2023). Cancer statistics, 2023. CA Cancer J Clin..

[62] Cheng Y., Ni Y.J., Tang L.M. (2023). ZNF521/EBF1 axis regulates AKR1B1 to promote the proliferation, migration, and invasion of gastric cancer cells. Kaohsiung J. Med. Sci..

[63] Shen S., Pu J., Lang B., McCaig C.D. (2011). A zinc finger protein Zfp521 directs neural differentiation and beyond. Stem Cell Res. Ther..

[64] Ohkubo N., Matsubara E., Yamanouchi J., Akazawa R., Aoto M., Suzuki Y., Sakai I., Abe T., Kiyonari H., Matsuda S. (2014). Abnormal behaviors and developmental disorder of hippocampus in zinc finger protein 521 (ZFP521) mutant mice. PLoS ONE.

[65] Mesuraca M., Galasso O., Leonardo G., Chiarella E., Scicchitano S., Vatrinet R., Morrone G., Bond H.M., Gasparini G. (2014). Expression profiling and functional implications of a set of zinc finger proteins, ZNF423, ZNF470, ZNF521 and ZNF780B, in primary osteoarthritic articular chondrocytes. Mediat. Inflamm..

[66] Hallis S.P., Kim J.M., Kwak M.K. (2023). Emerging Role of NRF2 Signaling in Cancer Stem Cell Phenotype. Mol. Cells.

[67] Lobry C., Oh P., Mansour M.R., Look A.T., Aifantis I. (2014). Notch signaling: Switching an oncogene to a tumor suppressor. Blood.

[68] Vilchez Mercedes S.A., Bocci F., Ahmed M., Eder I., Zhu N., Levine H., Onuchic J.N., Jolly M.K., Wong P.K. (2022). Nrf2 Modulates the Hybrid Epithelial/Mesenchymal Phenotype and Notch Signaling During Collective Cancer Migration. Front. Mol. Biosci..

[69] Sparaneo A., Fabrizio F.P., Muscarella L.A. (2016). Nrf2 and Notch Signaling in Lung Cancer: Near the Crossroad. Oxid. Med. Cell Longev..

[70] Wakabayashi N., Skoko J.J., Chartoumpekis D.V., Kimura S., Slocum S.L., Noda K., Palliyaguru D.L., Fujimuro M., Boley P.A., Tanaka Y. (2014). Notch-Nrf2 axis: Regulation of Nrf2 gene expression and cytoprotection by notch signaling. Mol. Cell Biol..

[71] Wakabayashi N., Chartoumpekis D.V., Kensler T.W. (2015). Crosstalk between Nrf2 and Notch signaling. Free Radic. Biol. Med..

[72] Kim J.H., Thimmulappa R.K., Kumar V., Cui W., Kumar S., Kombairaju P., Zhang H., Margolick J., Matsui W., Macvittie T. (2014). NRF2-mediated Notch pathway activation enhances hematopoietic reconstitution following myelosuppressive radiation. J. Clin. Investig..

[73] Murakami S., Shimizu R., Romeo P.H., Yamamoto M., Motohashi H. (2014). Keap1-Nrf2 system regulates cell fate determination of hematopoietic stem cells. Genes Cells.

[74] Wakabayashi N., Shin S., Slocum S.L., Agoston E.S., Wakabayashi J., Kwak M.K., Misra V., Biswal S., Yamamoto M., Kensler T.W. (2010). Regulation of notch1 signaling by nrf2: Implications for tissue regeneration. Sci. Signal..

[75] Morrison S.J., Perez S.E., Qiao Z., Verdi J.M., Hicks C., Weinmaster G., Anderson D.J. (2000). Transient Notch activation initiates an irreversible switch from neurogenesis to gliogenesis by neural crest stem cells. Cell.

[76] Taupin P., Gage F.H. (2002). Adult neurogenesis and neural stem cells of the central nervous system in mammals. J. Neurosci. Res..

[77] Hinoi E., Fujimori S., Wang L., Hojo H., Uno K., Yoneda Y. (2006). Nrf2 negatively regulates osteoblast differentiation via interfering with Runx2-dependent transcriptional activation. J. Biol. Chem..

[78] Hinoi E., Takarada T., Fujimori S., Wang L., Iemata M., Uno K., Yoneda Y. (2007). Nuclear factor E2 p45-related factor 2 negatively regulates chondrogenesis. Bone.

[79] Ganner A., Pfeiffer Z.C., Wingendorf L., Kreis S., Klein M., Walz G., Neumann-Haefelin E. (2020). The acetyltransferase p300 regulates NRF2 stability and localization. Biochem. Biophys. Res. Commun..

[80] Pouremamali F., Pouremamali A., Dadashpour M., Soozangar N., Jeddi N. (2022). An update of Nrf2 activators and inhibitors in cancer prevention/promotion. Cell Commun. Signal..

[81] Li X., Yan X., Wang Y., Kaur B., Han H., Yu J. (2023). The Notch signaling pathway: A potential target for cancer immunotherapy. J. Hematol. Oncol..

[82] Chiarella E., Codispoti B., Aloisio A., Cosentino E.G., Scicchitano S., Montalcini Y., Lico D., Morrone G., Mesuraca M., Bond H.M. (2020). Zoledronic acid inhibits the growth of leukemic MLL-AF9 transformed hematopoietic cells. Heliyon.

