# Supplementary figures and images for: Zinc Finger 521 Modulates the Nrf2-Notch Signaling Pathway in Human Ovarian Carcinoma
Source: Int J Mol Sci. 2023 Sep 29;24(19):14755. doi: 10.3390/ijms241914755 (PMC10572470; doi:10.3390/ijms241914755)

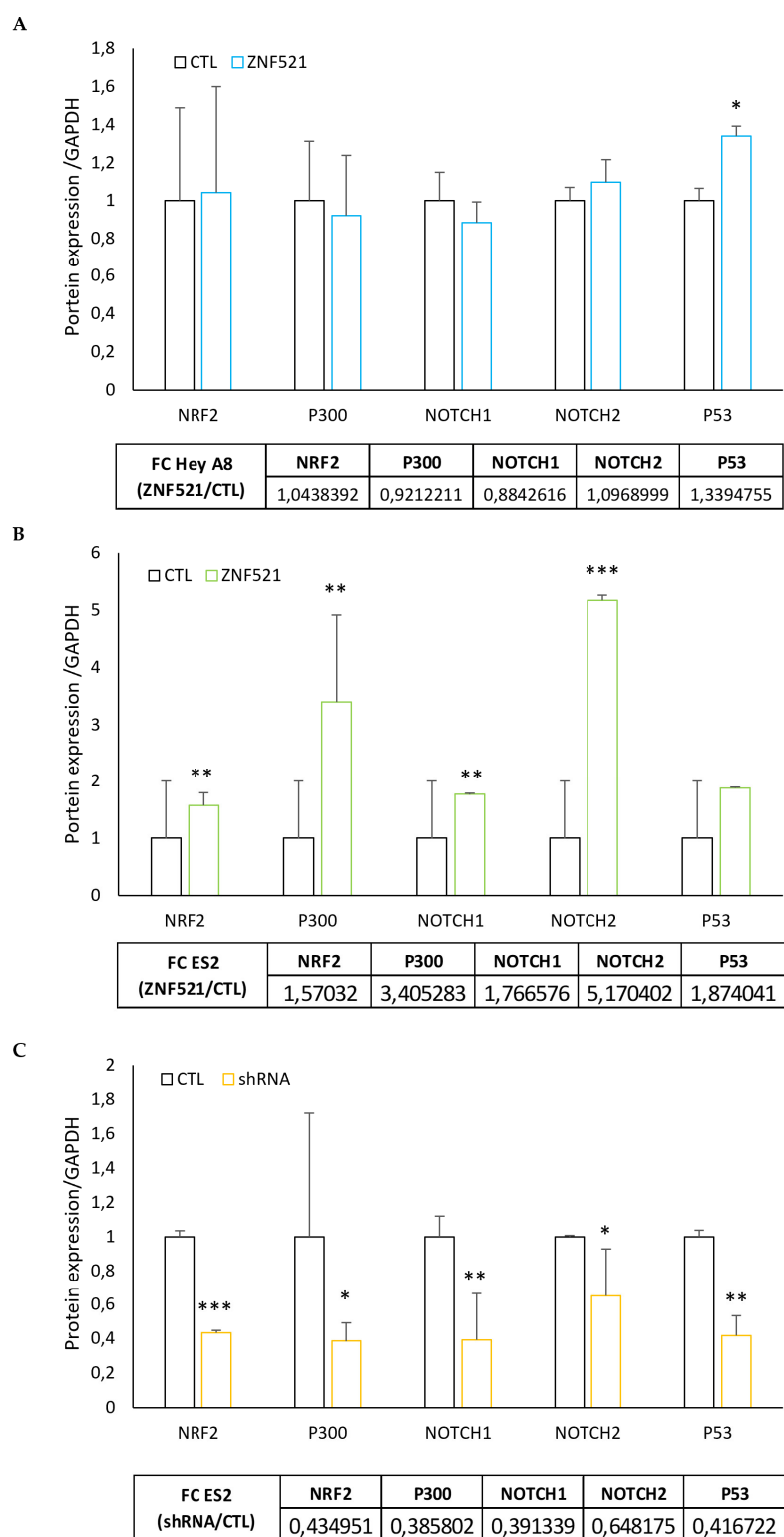

**Figure S1.** (A–C) Densitometric protein analysis: HeyA8 overexpressed for ZNF521.

Supplement: Supplementary file 1 [file ijms-24-14755-s001.zip › ijms-2609596-supplementary.pdf]
